# Supplementary material for: Broad Transcriptomic Impact of Sorafenib and Its Relation to the Antitumoral Properties in Liver Cancer Cells
Source: Cancers (Basel). 2022 Feb 25;14(5):1204. doi: 10.3390/cancers14051204 (PMC8909169; doi:10.3390/cancers14051204)
Supplement: Supplementary file 1 [file cancers-14-01204-s001.zip › cancers-1605103-supplementary figures.pdf]

cancers

MDPI

Supplementary material for article

Broad Transcriptomic Impact of Sorafenib and Its Relation to the Antitumoral Properties in Liver Cancer Cells

Laura Contreras [1,2], Alfonso Rodríguez-Gil [1,3,4], Jordi Muntané [1,4,5,*,†] and Jesús de la Cruz [1,2,*,†]

[1] Instituto de Biomedicina de Sevilla, Hospital Universitario Virgen del Rocío/CSIC/Universidad de Sevilla, E-41013, Seville, Spain. Contreras@us.es; arg@us.es; Jmuntane-ibis@us.es; Jdlcd@us.es
[2] Departamento de Genética, Facultad de Biología, E-41012, Universidad de Sevilla, Seville, Spain.
[3] Centro de Investigación Biomédica en Red de Cáncer (CIBERONC), E-28029, Madrid, Spain.
[4] Departamento de Fisiología Médica y Biofísica, Universidad de Sevilla, E-41009, Sevilla, Spain.
[5] Centro de Investigación Biomédica en Red de Enfermedades Hepáticas y Digestivas (CIBEREHD), E-28029, Madrid, Spain.
* Correspondence: Jmuntane-ibis@us.es (J.M.); Jdlcd@us.es (J.d.l.C.); Tel.: +34 955 923 122 (J.M.); +34 923 126 (J.d.l.C.).
† Both authors are equally responsible of this study.

Index

Table S1. List of DEGs in Sfb-treated HepG2 and SNU423 cells. **See supplementary Table S1 Excel file.**

Table S2. List of DEGs with log2(FC) higher than 1.5 or lower than -1.5 in Sfb-treated HepG2 and SNU423 cells**. See supplementary Table S2 Excel file.**

Table S3. List of pathways activated or inhibited upon a Sfb treatment in HepG2 cells. **See supplementary Table S3 Excel file.**

Figure S1. Quantitative RT-PCR validation of RNA-Seq data for HepG2 cells. A selection of down-regulated and up-regulated genes were assessed for gene expression through qPCR. Cells were grown for 24 h and then treated with 10 μM Sorafenib for 12 h before RNA extraction. The table compared the fold expression obtained by RNA-Seq *versus* the relative expression calculated by RT-PCR. Note that the corresponding mRNA levels quantified by RT-PCR from untreated cells (control) were arbitrarily set at 1.0. The *ACTB, BAX, BIRC3, CEBP, CPEB4, DUSP1, EIF4E2, EPOP, FEN1, GADD45B, IDI1, PCNA, SMAD7, TPI* and *VEGFA* genes were analysed. Expression levels were relativized to levels of 28S rRNA of each sample. Values are the mean ± S.D. values of at least three independent experiments performed in triplicate. Statistical significances were analysed by the Student's test (*$p < 0.05$, **$p < 0.01$, *** $p < 0.001$, ****$p < 0.0001$). Values for upregulated genes are shown in red, while those for downregulated genes are shown in green.

Figure S2. Quantitative RT-PCR validation of RNA-Seq data for SNU423 cells. A selection of down-regulated and up-regulated genes were assessed for gene expression through qPCR. Cells were grown for 24 h and then treated with 10 μM Sorafenib for 12 h before RNA extraction. The table compared the fold expression obtained by RNA-Seq *versus* the relative expression calculated by RT-PCR. Note that the corresponding mRNA levels quantified by RT-PCR from untreated cells (control) were arbitrarily set at 1.0. The *BIM, BOP1, BIRC3, CPEB4, DUSP1, EIF4E2, EPOP, GADD45B, IDI1, PHB, PCNA, SMAD7* and *TPI* genes were analysed. Expression levels were

relativized to levels of 28S rRNA of each sample. Values are the mean ± S.D. values of at least three independent experiments performed in triplicate. Statistical significances were analysed by the Student's test (*$p < 0.05$, **$p < 0.01$, ***$p < 0.001$, ****$p < 0.0001$). Values for upregulated genes are shown in red, while those for downregulated genes are shown in green.

Figure S3. Sorafenib lead to translation inhibition in HCC cells. Polysome profiles in HepG2 and SNU423 cells treated or not with Sorafenib (10 μM, 12 h). Cell extracts and polysome profile analysis were performed following the procedure described in Material and Methods. Ten A_{260} units of each extract were resolved in 7 to 50% sucrose gradients. The A_{254} was continuously monitored. Sedimentation is from left to right. The identity of the different peaks is indicated.

Figure S4. Cholesterol biosynthesis pathway in humans. This outline shows the cholesterol biosynthesis process with those enzymes whose genes were down-regulated by Sfb in red as suggested by our RNA-Seq analysis. The $\log_2$(FC) for each one is indicated in brackets.

Figure S5. Categories with opposite NES values in HepG2 and SNU423 cell lines. Reactome categories which show NES in opposite directions in the two cell lines analysed with the GSEA. NES and False Discovery Rate (FDR) *q*-Value are shown for each category. Panel (**A**) shows categories with FDR lower than 0.3 for HepG2 cells and its value in SNU423 cell line whereas panel (**B**) shows categories with FDR more significative for SNU423 cells and its respective value for HepG2 cells.

Figure S6. Uncropped Western Blots.

HepG2, validated genes

Genes	$Log_2(FC)$	RT-PCR
ACTB	1.27	1.66
BAX	-0.43	0.59
BIRC3	1.47	2.64
CEBP	1.80	1.94
CPEB4	1,95	4
DUSP1	1.73	9.84
EIF4E2	-1.34	0.63
EPOP	-2.56	0.25
FEN1	-2.10	0.38
GADD45B	3,01	15,1
IDI1	-2,50	0,11
PCNA	-1,47	0,17
SMAD7	1.88	1.33
TPI	-0.66	0.74
VEGFA	1.68	2,84

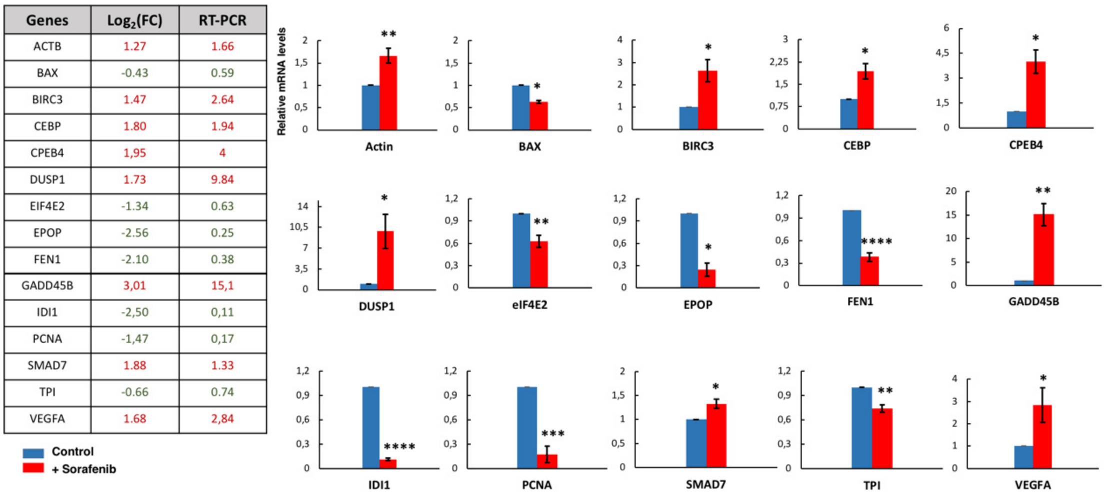

Figure S1. Quantitative RT-PCR validation of RNA-Seq data for HepG2 cells. A selection of down-regulated and up-regulated genes were assessed for gene expression through qPCR. Cells were grown for 24 h and then treated with 10 µM Sorafenib for 12 h before RNA extraction. The table compared the fold expression obtained by RNA-Seq *versus* the relative expression calculated by RT-PCR. Note that the corresponding mRNA levels quantified by RT-PCR from untreated cells (control) were arbitrarily set at 1.0. The *ACTB*, *BAX*, *BIRC3*, *CEBP*, *CPEB4*, *DUSP1*, *EIF4E2*, *EPOP*, *FEN1*, *GADD45B*, *IDI1*, *PCNA*, *SMAD7*, *TPI* and *VEGFA* genes were analysed. Expression levels were relativized to levels of 28S rRNA of each sample. Values are the mean ± S.D. values of at least three independent experiments performed in triplicate. Statistical significances were analysed by the Student's test (*p< 0.05, **p< 0.01, *** p< 0.001, ****p< 0.0001). Values for upregulated genes are shown in red, while those for downregulated genes are shown in green.

cancers

MDPI

SNU423, validated genes

Genes	$Log_2(FC)$	RT-PCR
BIM	1.70	1.29
BOP1	-0.84	0.69
BIRC3	2.20	2.64
CPEB4	2.01	3.50
DUSP1	1.28	4.27
EIF4E2	-1.1	0.69
EPOP	-1.62	0.25
GADD45B	2.72	27.4
IDI1	-2.21	0.11
PHB	-0.75	0.65
PCNA	-2.25	0.17
SMAD7	1.50	2.6
TPI	-0.9	0.53

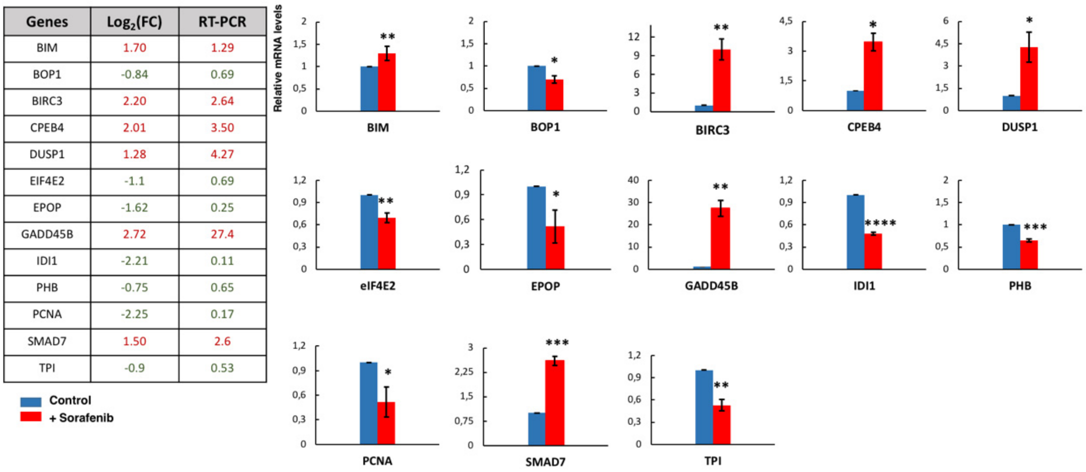

Figure S2. Quantitative RT-PCR validation of RNA-Seq data for SNU423 cells. A selection of down-regulated and up-regulated genes were assessed for gene expression through qPCR. Cells were grown for 24 h and then treated with 10 µM Sorafenib for 12 h before RNA extraction. The table compared the fold expression obtained by RNA-Seq *versus* the relative expression calculated by RT-PCR. Note that the corresponding mRNA levels quantified by RT-PCR from untreated cells (control) were arbitrarily set at 1.0. The *BIM, BOP1, BIRC3, CPEB4, DUSP1, EIF4E2, EPOP, GADD45B, IDI1, PHB, PCNA, SMAD7* and *TPI* genes were analysed. Expression levels were relativized to levels of 28S rRNA of each sample. Values are the mean ± S.D. values of at least three independent experiments performed in triplicate. Statistical significances were analysed by the Student's test (*$p < 0.05$, **$p < 0.01$, ***$p < 0.001$, ****$p < 0.0001$). Values for upregulated genes are shown in red, while those for downregulated genes are shown in green.

cancers

MDPI

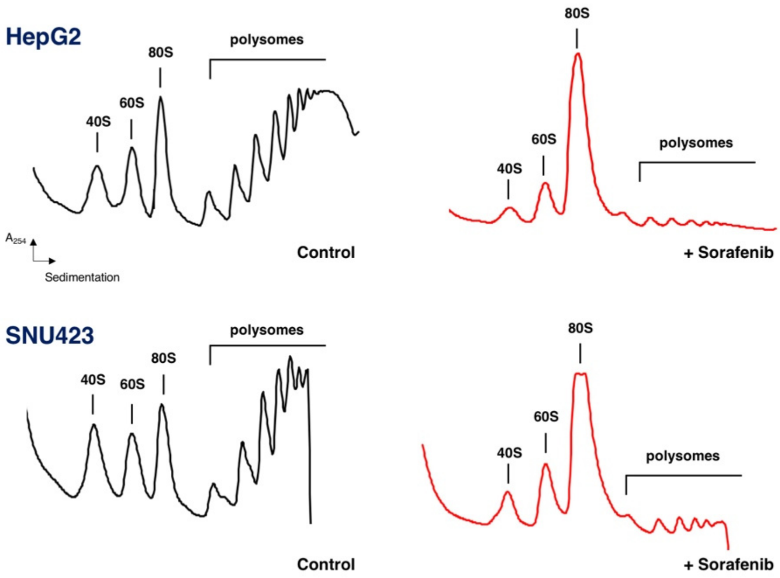

Figure S3. Sorafenib lead to translation inhibition in HCC cells. Polysome profiles in HepG2 and SNU423 cells treated or not with Sorafenib (10 μM, 12 h). Cell extracts and polysome profile analysis were performed following the procedure described in Material and Methods. Ten A_{260} units of each extract were resolved in 7 to 50% sucrose gradients. The A_{254} was continuously monitored. Sedimentation is from left to right. The identity of the different peaks is indicated.

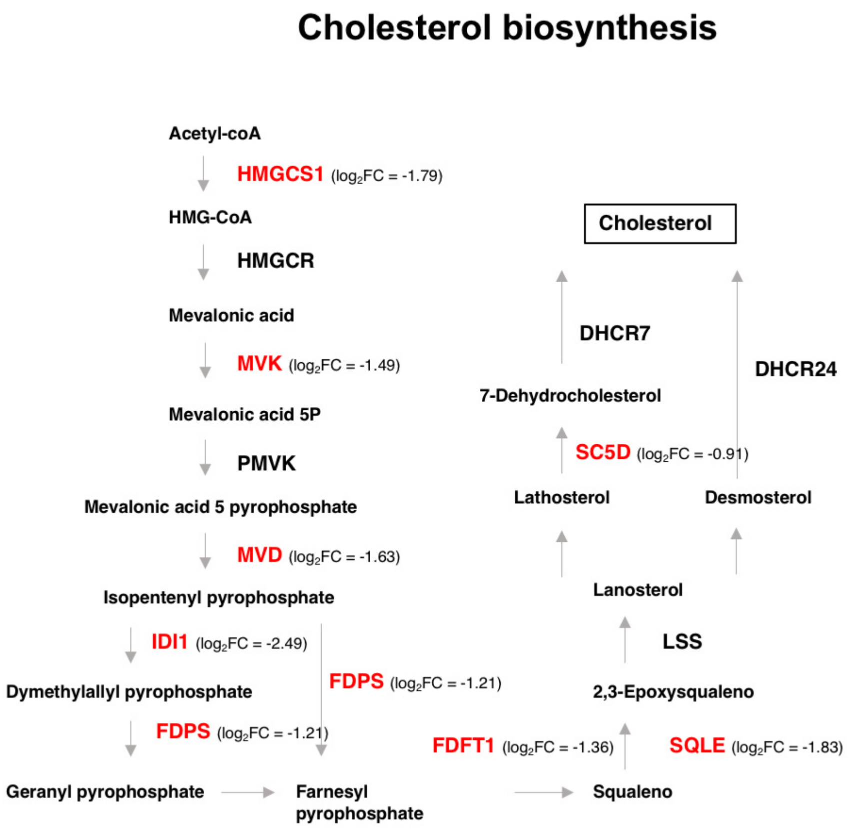

Figure S4. Cholesterol biosynthesis pathway in humans. This outline shows the cholesterol biosynthesis process with those enzymes whose genes were down-regulated by Sfb in red as suggested by our RNA-Seq analysis. The log_2(FC) for each one is indicated in brackets.

cancers

MDPI

A

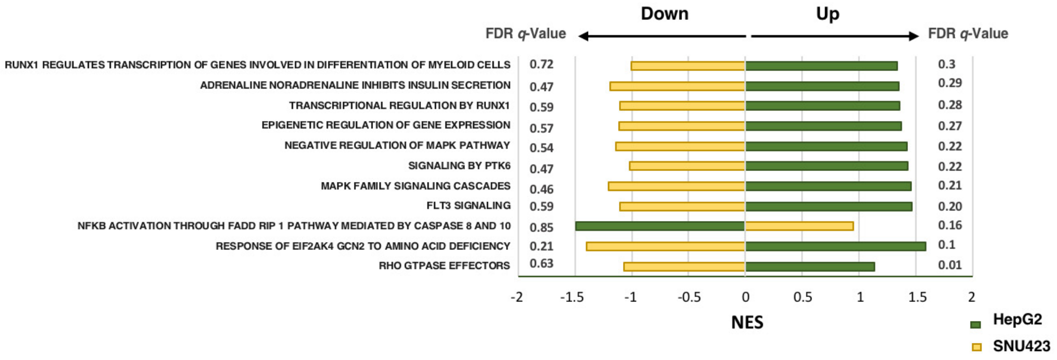

B

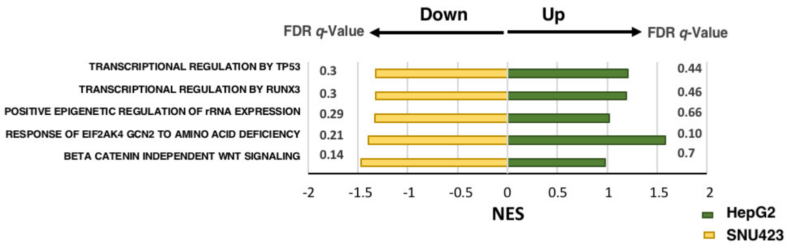

Figure S5. Categories with opposite NES values in HepG2 and SNU423 cell lines. Reactome categories which show NES in opposite directions in the two cell lines analysed with the GSEA. NES and False Discovery Rate (FDR) *q*-Value are shown for each category. Panel (**A**) shows categories with FDR lower than 0.3 for HepG2 cells and its value in SNU423 cell line whereas panel (**B**) shows categories with FDR more significative for SNU423 cells and its respective value for HepG2 cells.

Figure 6B. Uncropped western blot for the NDUFS1 image

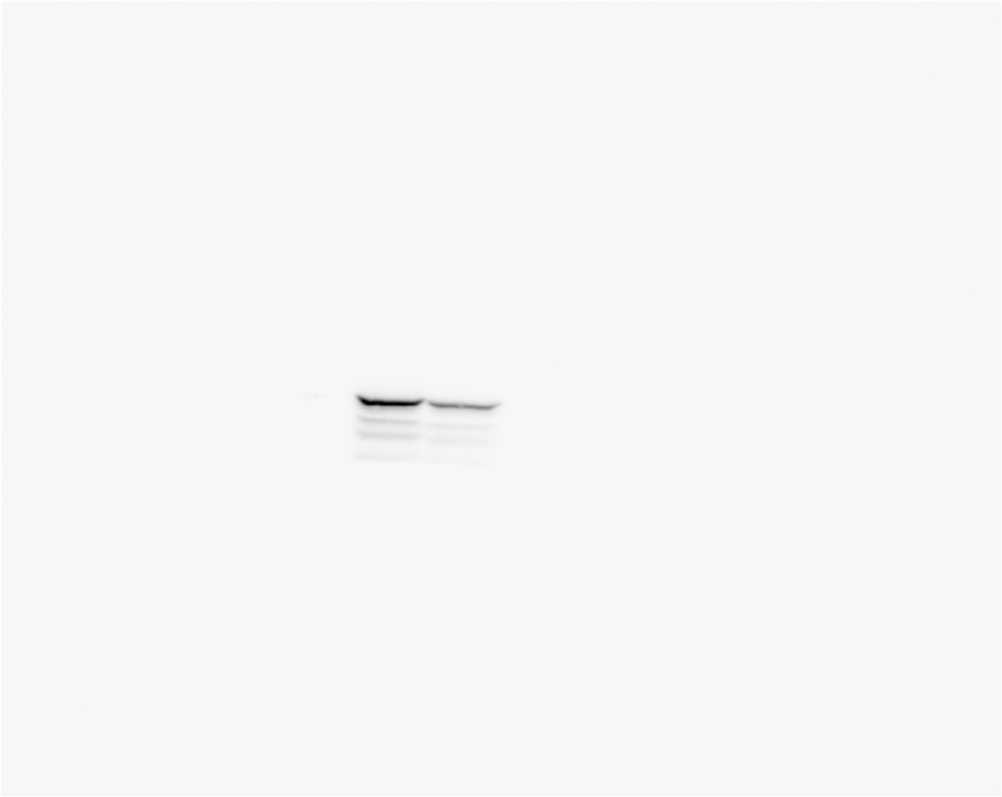

cancers

MDPI

Figure 6B. Uncropped western blot for the NDUFS2 image (note that the bands of interest are the ones at the bottom of the gel)

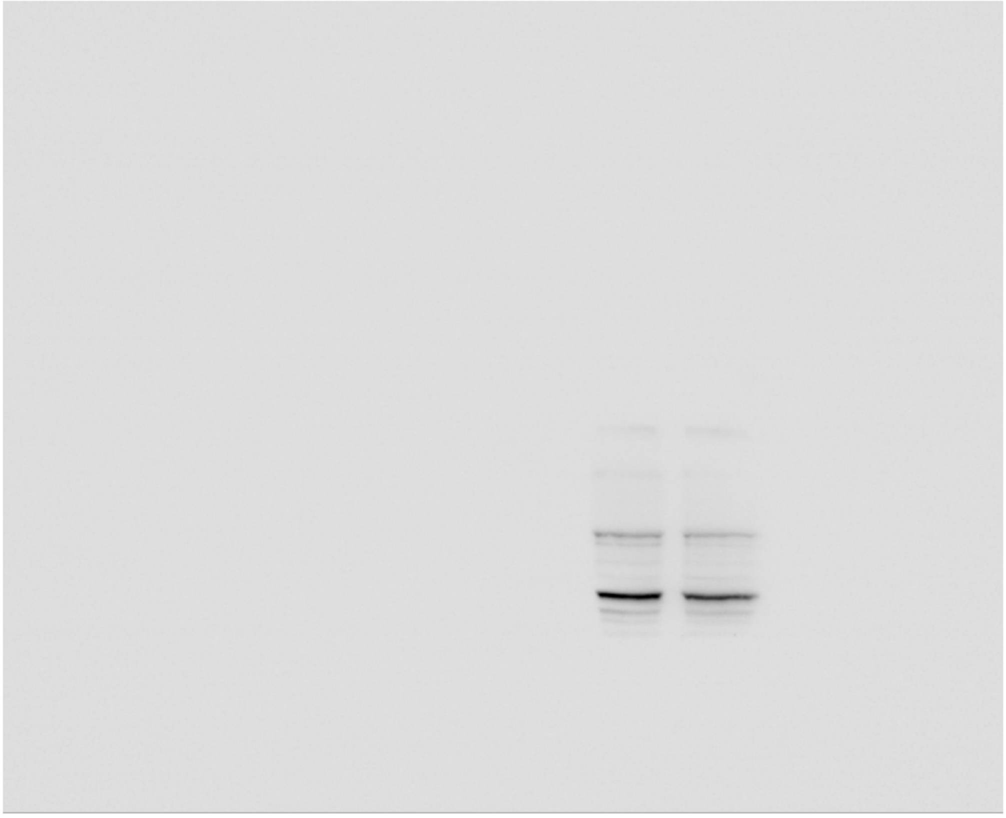

cancers

MDPI

Figure 6B. Uncropped western blot for the NDUFV2 image

cancers

MDPI

Figure 6B. Uncropped western blot for the GAPDH image (note that the bands of interest are the ones at the bottom of the gel)

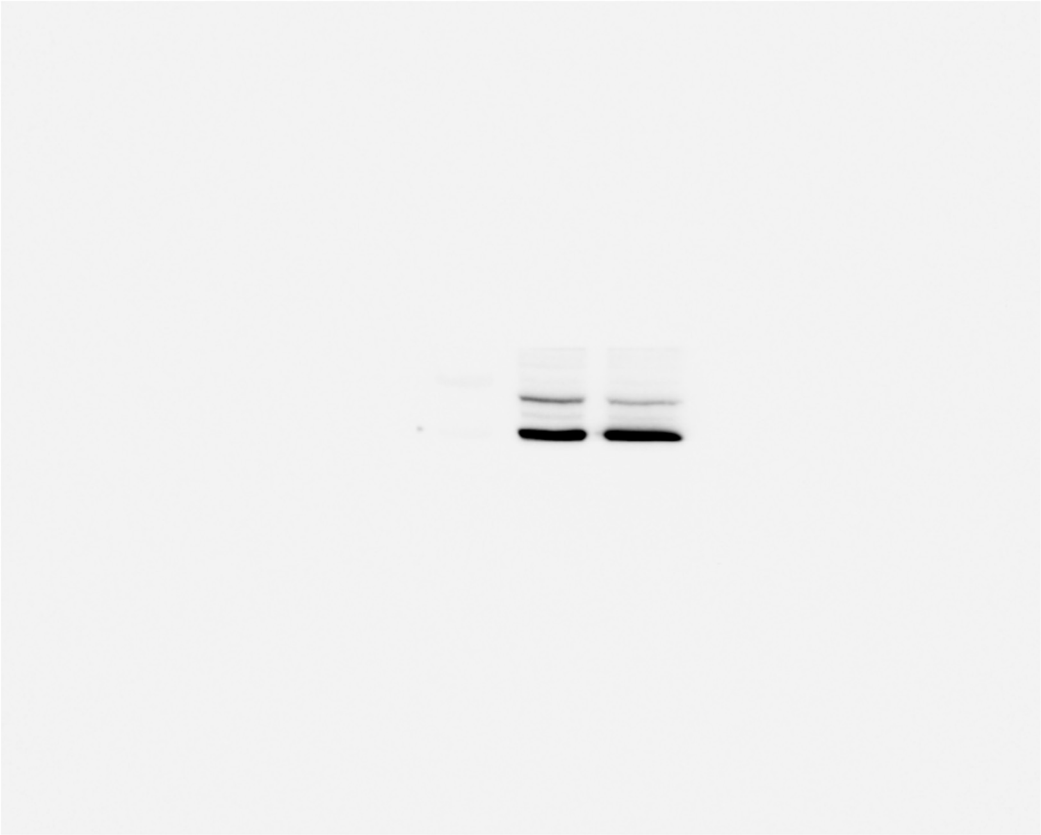

Figure S6. Uncropped Western Blots.
